# Reagent and Data Resources for Investigation of RNA Binding Protein Functions in *Drosophila melanogaster* Cultured Cells

**DOI:** 10.1534/g3.115.019364

**Published:** 2015-07-21

**Authors:** Stephanie E. Mohr, Yanhui Hu, Kirstin Rudd, Michael Buckner, Quentin Gilly, Blake Foster, Katarzyna Sierzputowska, Aram Comjean, Bing Ye, Norbert Perrimon

**Affiliations:** * Drosophila RNAi Screening Center, Department of Genetics, Harvard Medical School, Boston, Massachusetts 02115; † Life Sciences Institute, University of Michigan, Ann Arbor, Michigan 48109; ‡ Howard Hughes Medical Institute, Boston, Massachusetts 02115

**Keywords:** *Drosophila*, RNA binding protein, RNAi, data sharing, high-throughput screen

## Abstract

RNA binding proteins (RBPs) are involved in many cellular functions. To facilitate functional characterization of RBPs, we generated an RNA interference (RNAi) library for *Drosophila* cell-based screens comprising reagents targeting known or putative RBPs. To test the quality of the library and provide a baseline analysis of the effects of the RNAi reagents on viability, we screened the library using a total ATP assay and high-throughput imaging in *Drosophila* S2R+ cultured cells. The results are consistent with production of a high-quality library that will be useful for functional genomics studies using other assays. Altogether, we provide resources in the form of an initial curated list of *Drosophila* RBPs; an RNAi screening library we expect to be used with additional assays that address more specific biological questions; and total ATP and image data useful for comparison of those additional assay results with fundamental information such as effects of a given reagent in the library on cell viability. Importantly, we make the baseline data, including more than 200,000 images, easily accessible online.

High-throughput cell-based RNA interference (RNAi) screening, including in *Drosophila* cultured cells, provides a format for large-scale interrogation of gene function. RNAi technology has significantly improved over time and the approach now serves as a robust method for functional genomics discovery (Mohr *et al.* 2014). Many advances have helped limit false discovery (*i.e.*, false-positive and false-negative results) in high-throughput screens in general and RNAi screens specifically. Nevertheless, RNAi screens remain associated to some degree with false discovery, including sequence-specific off-target effects (OTEs) (Kulkarni *et al.* 2006; Moffat *et al.* 2007). Additional factors that impact false discovery and thus affect overall primary screen data quality include the quality of gene annotations used for RNAi reagent design, the rules and filters applied to design of the reagents, the number of unique reagents per gene in the primary screening library, the layout of control and experimental reagents on screen assay plates, the number of replicates performed in a given screen, and the types of analyses performed with the primary data (Birmingham *et al.* 2009; Boutros and Ahringer 2008; DasGupta *et al.* 2007; Mohr *et al.* 2014) .

For some projects, it is more appropriate to screen a subset of genes rather than to perform a genome-wide screen (Boutros and Ahringer 2008). A focused approach can reduce costs, allow for rapid follow-up on results from other omics studies, provide focus to follow-up assays, help establish positive controls (*i.e*., when done prior to a full-genome screen), and/or help address false-negative discovery (*i.e*., when done after a full-genome screen). In addition, when the target gene number is reduced, it becomes easier to include more unique reagents per gene, include a generous number of positive and negative controls, and apply optimal plate layouts (*e.g* ., avoid the use of edge wells, which are subject to position-dependent effects or "edge effects") (Boutros and Ahringer 2008; Mohr 2014). Potential disadvantages of screening with a focused library include missing unexpected results and the fact that gene set enrichment analyses, which can be highly informative when applied to genome-wide screen data, might not be as informative or appropriate because the initial library is biased.

The usefulness of a library, small or large, improves when researchers know *a priori* which reagents in the library affect general cell health (*e.g.*, compromise cell division, growth, and/or viability), because this can impact interpretation of results obtained with more sophisticated assays (DasGupta *et al.* 2007). Thus, obtaining "baseline" data and making these data freely available are important for establishing a reagent library as a high-quality and useful resource. Additional factors that influence the usefulness and quality of an RNAi reagent library can include the number of unique reagents per gene and effectiveness of the reagents.

RNA binding proteins (RBPs) are of special interest because they are involved in a broad range of fundamental cellular activities, including RNA editing, localization, stability, translation, transcription, and transport (Gamberi *et al.* 2006; Glisovic *et al.* 2008) as well as DNA damage responses (Dutertre *et al.* 2014). RBPs also regulate cellular processes such as pluripotency (Ye and Blelloch 2014) and senescence (Wang 2012), and have demonstrated relevance to human diseases, including inherited diseases, neuronal diseases, and cancer (Castello *et al.* 2013; Fredericks *et al.* 2015; Gerstberger *et al.* 2014; Kim *et al.* 2009; Lenzken *et al.* 2014). Notably, many of these processes can be studied using cultured cell assays, making the gene set particularly relevant for development of a high-quality cell-based RNAi screen library. We curated a list of genes that encode RBPs, synthesized a corresponding *Drosophila* cell-based RNAi reagent library, performed an initial characterization of the library, and, importantly, made raw and analyzed data from this initial characterization, including more than 100,000 images, available online. Thus, studies utilizing this resource should help our understanding of RBPs in *Drosophila* and other species.

## Materials and Methods

### Bioinformatics analysis and curation of *Drosophila melanogaster* RBPs

We generated a list of known and putative *Drosophila* RBPs using a combination of literature and database mining and expert curation. The major resources we explored are gene ontology annotation (http://www.ncbi.nlm.nih.gov/) (Harris *et al.* 2004), UniProt protein annotation (http://www.uniprot.org/) (Apweiler *et al.* 2004), and protein domain annotation of InterPro (http://www.ebi.ac.uk/interpro/) (Apweiler *et al.* 2001). The target gene list contains 427 unique genes that code for proteins binding to mRNA, rRNA, tRNA, and other noncoding RNAs.

### *In vitro* synthesis of dsRNA for RNAi

We used UP-TORR (http://flyrnai.org/up-torr) (Hu *et al.* 2013) to identify appropriate reagent templates in our collection. As needed for two reagents per gene coverage, we designed additional dsRNAs using SnapDragon (http://www.flyrnai.org/cgi-bin/RNAi_find_primers.pl). We used standard protocols to prepare dsRNAs (Mohr 2014; see http://www.flyrnai.org/DRSC-PRR.html for protocol). In brief, we used liquid handling automation to individually select PCR templates for double-stranded RNA (dsRNA) synthesis based on our existing collections. Next, we quality-analyzed the dsRNAs, used a Multiprobe liquid handling robot (PerkinElmer) to normalize dsRNA concentrations, and used an Agilent Bravo liquid handling robot to array normalized dsRNAs into a final 384-well "assay-ready" format.

### Cell culture and RNAi knockdown

All studies were performed using S2R+ cultured cells and standard methods for culture. Cells and reagents were dispensed into 384-well assay plates using a WellMate (Thermo) or Mantis (Formulatrix) liquid handling robot. For RNAi, cells were dispensed into assay plates containing dsRNAs at a standard concentration for the "bathing" method (passive uptake; see http://www.flyrnai.org/DRSC-PRR.html for a step-by-step protocol).

### High-throughput screening

We used the Cell Titer Glo reagent (Promega) and a Spectramax Paradigm automated luminometer (Molecular Devices) to measure total well levels of ATP following RNAi knockdown (protocol at http://www.flyrnai.org/DRSC-Protocol-atp-levels.html). The data were analyzed using standard statistical approaches. For imaging, we used an Opera automated confocal imaging system (PerkinElmer) to image cells stained with DAPI and FITC-Phalloidin following RNAi knockdown (http://www.flyrnai.org/DRSC-Protocol-cellfix.html for protocol). Images were obtained using 20× or 60× water immersion lenses and confocal fluorescence imaging.

### Data availability

**Figure 1:**
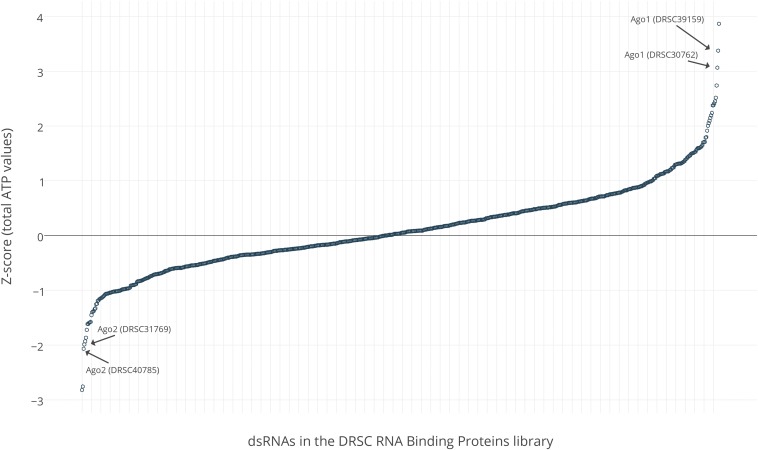
Distribution of Z-scores from a primary screen using total ATP levels as an assay readout with a dsRNA library targeting genes that encode known or putative RBPs. The two dsRNAs in the library that target *AGO2* or *AGO1* are indicated with arrows. A summary of hits from the screen is presented in Table 1, Table 2, and Table 3. A view of the graph that is dynamic (*e.g.*, gene symbols appear when the user hovers on specific data points) as well as the underlying data are available at Plotly (https://plot.ly/∼semohr/60). The raw and analyzed data are also available at the DRSC FlyRNAi database (http://www.flyrnai.org/DRSC-RBP_data.php) and at NCBI PubChem BioAssays (https://pubchem.ncbi.nlm.nih.gov/assay/assay.cgi?aid=1159508).

Raw and analyzed data from the total ATP screen assay are available for view or download at our FlyRNAi.org web site (Flockhart *et al.* 2012). Specifically, data can be exported from FlyRNAi as comma-separated values (CSV) files or searched and viewed online using the Gene Lookup or Screen Summary graphical user interfaces at www.flyrnai.org (Flockhart *et al.* 2012). To create the visualization of the total ATP data set shown in Figure 1, we exported the raw and analyzed data from our database, uploaded the data to Plotly (https://plot.ly/), and used tools at Plotly to generate the scatter plot and labels. An interactive view of the graph of Z-scores shown in Figure 1 as well as the underlying data are freely available at Plotly (https://plot.ly/∼semohr/60). The data are also available at PubChem BioAssays https://pubchem.ncbi.nlm.nih.gov/assay/assay.cgi?aid=1159508. Data files from the PerkinElmer Opera confocal microscope are captured in the proprietary FLEX file format. Images were converted from FLEX format to TIFF format using Acapella software. Raw images (.flex) or minimally processed images (.tiff) are available on request for automated analysis. For online search and view, we uploaded scaled TIFF image files at Flickr (Yahoo, Inc.), where they are made available in JPEG format. The images were made public under a Creative Commons license. They are associated with the user account “drsc_lab” and can be viewed at https://www.flickr.com/photos/132735911@N05/. The Opera image file naming system separates the following with an underscore: plate ID; well location; position within the well; channel; and position in the Z-axis. For example, the file 100B225_F24_S25_W1_Z1 corresponds to plate 100B225 (“100” indicates the test plate layout; “B” indicates that dsRNAs were present at the concentration appropriate for the bathing method of dsRNA uptake; and “225” reflects the aliquot number for the specific plate used), well F24, position S25, and channel W1 (in this case, FITC-Phalloidin staining) at a single plane of focus (Z1). The “gene list” file for the RBP library downloadable at http://www.flyrnai.org/DRSC-SUB.html can be used to associate specific plate-wells with specific DRSC reagents and target genes. A searchable lookup table linking specific reagents and genes to image data at Flickr is available at http://www.flyrnai.org/DRSC-RBP_data.php. At the Flickr account, subsets of control images have been organized into “albums” (folders). We also assigned some key words to all images (*e.g.*, “confocal”) or subsets of images (*e.g.*, “hoip”), and others were automatically assigned by Flickr (*e.g.*, “blackandwhite”).

## Results and Discussion

### A *Drosophila* RNAi library targeting RNA binding domain-containing proteins

The *Drosophila* RNAi Screening Center (DRSC) and others have developed robust approaches for RNAi reagent and library design (Mohr 2014)). We applied these approaches to production of a focused library targeting genes that encode RNA binding domain-containing proteins. To do this, we first generated a list of known and predicted *Drosophila* RBPs using a combination of literature and database mining and expert curation, and then generated the reagent library (see *Materials and Methods*). Features of the dsRNA library include: design of dsRNAs with minimal predicted OTEs using SnapDragon (http://www.flyrnai.org/snapdragon); coverage with two or more unique dsRNAs per gene; tracking of dsRNA production and quality analysis in our database; and inclusion of standard dsRNA controls on each 384-well assay plate. To minimize edge effects (*i.e.*, reduced viability or other data anomalies frequently observed at assay plate edges), dsRNAs were excluded from the outermost two wells of each plate. We included two positive controls: dsRNA targeting *thread (Diap1*), which results in cell death, and dsRNA targeting *Rho1*, which leads to the appearance of large, binucleate cells. We also included three negative controls, dsRNAs targeting *GFP* and *LacZ*, neither of which is present in wild-type *Drosophila* cells, and wells with an equal volume of water (no dsRNA). The layout of control dsRNAs on each plate can be viewed at http://www.flyrnai.org/DRSC-LAY.html and the layout of all dsRNAs in the library can be downloaded from http://www.flyrnai.org/DRSC-SUB.html.

### Screen for changes in total ATP levels using the RBPs library

We next used Promega Cell Titer Glo to measure total ATP levels after 5 days of incubation of S2R+ cultured cells in three replicates of each unique assay plate in the library. Full raw and analyzed data are available at the DRSC FlyRNAi.org database (Flockhart *et al.* 2012). The distribution of Z-scores for dsRNAs in the screen is shown in Figure 1. An interactive view of the graph as well as the data are freely available at https://plot.ly/∼semohr/60. Genes that might be considered primary screen "hits" (positive results) from the total ATP levels screen based on commonly applied criteria are summarized in Table 1, Table 2, and Table 3. By Z-score, the strongest hit for "high ATP levels" is *Argonaute-1* (*AGO1*), an Argonaut family protein required for miRNA maturation (Okamura *et al.* 2004). The strongest hit for "low ATP levels" is *hoi-palloi* (*hoip*), which encodes a ribosomal protein and has been associated with neuronal mutant phenotypes in *Drosophila* (Prokopenko *et al.* 2000). The opposing results obtained with dsRNAs targeting *AGO1* and *AGO2* (Table 1) are consistent with the distinct roles of AGO1 and AGO2 in small RNA biogenesis (Okamura *et al.* 2004; Okamura and Lai 2008; Zhou *et al.* 2008). The result with *AGO1* suggests that one or more microRNAs (miRNAs) might normally dampen growth and/or viability of S2R+ cells, consistent with roles identified for some miRNAs in signaling and cancer (Hagen and Lai 2008; Jackstadt and Hermeking 2015). In the case of *AGO2*, suppression of the AGO2-mediated endogenous small interfering RNA (siRNA) pathway might result in increased mobilization of transposons (Czech *et al.* 2008), perhaps accounting for the observed compromise in cell viability, and/or might result in detrimental changes to metabolism or stress responses (Lim *et al.* 2011).

**Table 1:** Genes for which two of two unique dsRNA designs each confer good statistical significance (Z-score ≥1.5 or ≤−1.5 for 2 of 2 dsRNAs) in the total ATP levels assay

FlyBase ID	Gene Symbol	DRSC Reagent ID	Z-score	Expressed*^a^*
FBgn0262739	*AGO1*	DRSC30762	3.06	Yes
		DRSC39159	3.38	
FBgn0027873	*Cpsf100*	DRSC34503	1.60	Yes
		DRSC14146	1.71	
FBgn0016978	*snRNP-U1-70K*	DRSC28803	1.79	Yes
		DRSC34940	2.05	
FBgn0087035	*AGO2*	DRSC40785	−2.07	Yes
		DRSC31769	−1.86	
FBgn0015393	*hoip*	DRSC03546	−2.82	Yes
		DRSC31857	−2.75	

a Evidence of expression in untreated S2R+ cells based on modENCODE data and queried using the DRSC cell expression tool http://www.flyrnai.org/cellexpress.

**Table 2:** Genes in addition to those shown in Table 1 for which the average Z-score values of the two unique dsRNA designs pass a threshold Z-score of ≥1.5 or ≤−1.5

FlyBase ID	Gene Symbol	DRSC Reagent ID	Individual Z-scores	Average Z-score	Expressed*^a^*
FBgn0035947	*Srp68*	DRSC36443	−0.01	1.93	Yes
		DRSC28655	3.87		
FBgn0003575	*su(s)*	DRSC18839	1.12	1.56	Yes
		DRSC34942	2.00		
FBgn0011666	*Msi*	DRSC34282	1.19	1.96	Yes
		DRSC17003	2.74		
FBgn0028577	*pUf68*	DRSC25143	−1.93	−1.51	Yes
		DRSC31973	−1.10		
FBgn0015818	*Spx*	DRSC23542	−1.99	−1.50	Yes
		DRSC31760	−1.01		

a Evidence of expression in untreated S2R+ cells based on modENCODE data and queried using the DRSC cell expression tool http://www.flyrnai.org/cellexpress.

**Table 3:** Genes in addition to those shown in Table 1 and Table 2 for which one of the two dsRNAs confers a high Z-score (≥1.5 or ≤−1.5) in the total ATP levels assay

FlyBase ID	Gene Symbol	DRSC Reagent ID	Z-score	Expressed*^a^*
FBgn0052707	*APC4*	DRSC35244	2.09	Yes
FBgn0011566	*Bzd*	DRSC38965	2.52	Yes
FBgn0035872	*CG7185*	DRSC34893	2.45	Yes
FBgn0036534	*DCP2*	DRSC10597	2.24	Yes
FBgn0000559	*EF2*	DRSC32107	2.41	Yes
FBgn0020443	*Elf*	DRSC03322	2.38	Yes
FBgn0051992	*gw*	DRSC17135	2.19	Yes
FBgn0004401	*Pep*	DRSC11252	2.38	Yes
FBgn0003612	*Su(var)2-10*	DRSC33201	2.15	Yes
FBgn0033741	*CG8545*	DRSC35557	1.91	Yes
FBgn0028982	*Spt6*	DRSC31075	1.8	Yes
FBgn0264962	*Inr-a*	DRSC34272	1.71	Yes
FBgn0035831	*CG8038*	DRSC29825	1.7	Yes
FBgn0015778	*rin*	DRSC33099	1.66	Yes
FBgn0003520	*stau*	DRSC34478	1.63	Yes
FBgn0051957	*CG31957*	DRSC00207	1.62	Yes
FBgn0038934	*Gld2*	DRSC23037	1.61	No
FBgn0039920	*CG11360*	DRSC17124	1.6	Yes
FBgn0026086	*Adar*	DRSC42388	1.6	Yes
FBgn0027841	*CstF-64*	DRSC29486	1.58	Yes
FBgn0036974	*eRF1*	DRSC27974	1.56	Yes
FBgn0042712	*HBS1*	DRSC35780	1.53	Yes
FBgn0014870	*Psi*	DRSC34932	1.52	Yes
FBgn0028474	*CG4119*	DRSC27945	1.51	Yes
FBgn0029880	*CG14443*	DRSC17936	1.51	No
FBgn0040365	*CG14628*	DRSC18626	1.5	Yes
FBgn0026722	*drosha*	DRSC35708	1.5	Yes
FBgn0033060	*CG7849*	DRSC26457	−1.58	Yes
FBgn0039229	*Saf-B*	DRSC16167	−1.58	Yes
FBgn0259935	*CG42458*	DRSC26691	−1.59	No
FBgn0266917	*CG16941*	DRSC15166	−1.61	Yes
FBgn0010488	*NAT1*	DRSC30053	−1.61	Yes
FBgn0039175	*beta-PheRS*	DRSC41194	−1.72	Yes

a Evidence of expression in untreated S2R+ cells based on modENCODE data and queried using the DRSC cell expression tool http://www.flyrnai.org/cellexpress

Table 1 comprises a conservative list of high-confidence hits; Table 2 and Table 3 add additional lower-confidence hits that might be interrogated in follow-up studies and/or compared with other screen data. Each of the reagents in Table 3 has no predicted off-targets and is predicted to target all isoforms of the gene (Hu *et al.* 2013). Moreover, modENCODE cell expression data for S2R+ cultured cells suggest that most of these genes are normally expressed in S2R+ (Booker *et al.* 2011; Lee *et al.* 2014). Thus, we predict that most of these are "single hits" due to a false-negative result with the other dsRNAs targeting the same genes rather than due to false-positive discovery with the positive dsRNA. To further improve library quality, we plan to add additional unique dsRNAs for those cases in which two different dsRNAs targeting the same gene do not give comparable results (*i.e.*, for a subset of genes in Table 2 and all genes in Table 3). The complete set of Z-scores as well as modENCODE results regarding expression in S2R+ cells (Booker *et al.* 2011; Lee *et al.* 2014) are available in supporting information, Table S1. Moreover, we have made raw and analyzed data available via a variety of online repositories as summarized at http://www.flyrnai.org/DRSC-RBP_data.php.

### High-throughput imaging using the RBPs library

To further characterize the RBP library, we stained cells with DAPI and FITC fluorescence-conjugated Phalloidin to visualize nuclei and filamentous actin, respectively, in paraformaldehyde-fixed S2R+ cells. The cells were then imaged at 20× and 60× using a fluorescence confocal imaging system. As expected, *Rho1* serves as an effective positive control for image-based assays, as treatment with *Rho1* dsRNA results in a binuclear phenotype readily detectable using a DNA dye and any marker that defines the cell body. These cells also appear larger. Also as expected, few cells are detected in images corresponding to *thread (Diap1)* dsRNA control wells. Consistent with the total ATP assay results (Table 1), few cells are detected in images corresponding to wells treated with dsRNA targeting *AGO2* or *hoip*.

Availability of RNAi screen data in the form of text or numbers is facilitated by public databases including FlyRNAi, GenomeRNAi, and NCBI PubChem BioAssays (Flockhart *et al.* 2012; Gilsdorf *et al.* 2010; Wang *et al.* 2014). Ideally, researchers will also be provided with access to baseline image data, *e.g.*, to help identify reagents with gross effects on cell morphology. However, high-throughput, high-content image data sets are large both in total size and in terms of the total number of individual images, presenting significant challenges to image data management. As a result, making images easily available for search and view online has been seen as a significant challenge. Although solutions arising from within the biological community such as the Online Microscopy Environment OMERO platform (Allan *et al.* 2012) hold strong promise for making image data public online, the solutions offered to date are not easy to implement and support, requiring significant expertise and infrastructure. As a result, although it is relatively easy to share complete sets of screen image data, such as for automated analyses (*e.g.*, because an entire image data set can be shared using a file transfer protocol or on an external hard drive), making it possible for researchers to easily view images associated with a specific subset of reagents has remained a barrier. To find a near-term, feasible solution to making a set of baseline image data publically searchable and viewable online, we chose to make images available at flickr.com. Advantages of Flickr include that the site has been around for more than 10 years, it offers 1 terabyte of free image storage, and it allows sharing of images under a Creative Commons license agreement, the same type of agreement used by open access journals. More than 200,000 images have been deposited with the user account drsc_lab at Flickr.com and can be viewed at https://www.flickr.com/photos/132735911@N05/. For quick reference, example images from wells treated with dsRNAs targeting *Rho1*, *thread (Diap1)*, *AGO2*, or *hoip* are organized as “albums” within the drsc_lab collection (https://www.flickr.com/photos/132735911@N05/albums). In addition, a searchable lookup table is available at http://www.flyrnai.org/DRSC-RBP_data.php. For additional information on how to navigate the image data resource see *Data availability* in *Materials and Methods*.

## Conclusion

The DRSC has a longstanding commitment to building high-quality functional genomics reagents and making large-scale screen data publically available through its own database, FlyRNAi (Flockhart *et al.* 2012), as well as at meta-databases such as GenomeRNAi (Gilsdorf *et al.* 2010) and PubChem BioAssays (Wang *et al.* 2014). Here, we have shown that established and new media solutions can be utilized effectively for public data availability. The baseline data sets for the RBPs library help provide a measure of overall library quality and will aid the interpretation of results obtained using other assays. The DRSC has and continues to generate other focused libraries based on community input. Analysis of other libraries to the same extent as that presented in this study would add value to existing and new libraries. Moreover, because a large proportion of the genes represented in the RBP and other DRSC focused libraries have been conserved, the results of screens using these libraries are likely to have impact beyond *Drosophila*.

## Supplementary Material

Supporting Information
