# Supplementary material for: Reagent and Data Resources for Investigation of RNA Binding Protein Functions in Drosophila melanogaster Cultured Cells
Source: G3 (Bethesda). 2015 Jul 21;5(9):1919–24. doi: 10.1534/g3.115.019364 (PMC4555228; doi:10.1534/g3.115.019364)
Supplement: Supporting Information [file supp_5_9_1919__index.html]

Reagent and Data Resources for Investigation of RNA Binding Protein Functions in Drosophila melanogaster Cultured Cells — Supporting Information 

# Reagent and Data Resources for Investigation of RNA Binding Protein Functions in *Drosophila melanogaster* Cultured Cells

## Supporting Information for Mohr *et al.*, 2015

**Files in this Data Supplement:**

- Table S1 - Complete set of Z-scores and modENCODE results regarding expression in S2R+ cells. (.xlsx, 52 KB)
